# Notes and News

**Published:** 1897-07-10

**Authors:** 


### Notes and News.

(Collected and Communicated.)

The honour conferred upon Dr. Haffkine and the definite salary granted to him has given much satisfaction, as showing due appreciation of valuable research.

The ceremony of laying the foundation-stone of the new cancer wing of the Middlesex Hospital, originally fixed for July 1st, is to take place on July 15th at 5 p.m.

The Honorary Secretaries of the Prince of Wales's Hospital Fund have received the munificent sum of £5,512 15s. 6d. from the Freemasons of England as a contribution to the fund, in commemoration of the Queen's reign.

The treasurer of Guy's Hospital has received a Jubilee gift of £3,000 towards the re-endowment fund from a lady who desires to remain anonymous. The interest on this sum will be applied towards the maintenance of beds in Queen Victoria Ward, recently reopened by the Prince of Wales.

The *Medical Record* of New York says that the Osteopathy Bill passed by the Illinois Legislature has been vetoed by the Governor of that State. This was a Bill to give legal sanction to the practice of bone-setting by unqualified practitioners. It is very satisfactory to find that the Governor has taken a common-sense view of the matter.

Although the presence of a certain amount of leprosy is well known in Russia, it has not so far caused any grave anxiety. Several cases of leprosy have, however, appeared in a district hitherto exempt, causing some excitement amongst the inhabitants. The cases have appeared in Kuban and Don Cossack, where serious measures are being taken by the authorities to check the progress of the disease.

Mr. H. Roberts, who entertained the seventy survivors of Balaclava on Jubilee Day, has, upon inquiry into their circumstances, ascertained that 20 of these old soldiers are in very needy circumstances; he therefore proposes to collect subscriptions towards placing them in comfort for the rest of their lives. He states himself ready to pay all incidental expenses that the whole money subscribed may reach the men. He will be glad to receive contributions at 158, Fleet Street, E.C.

It appears likely that Marseilles will succeed in establishing a university of its own.

The Duke and Duchess of York have consented to be present at the official destruction of the plates from which the Hospital Stamps have been printed, and have fixed Friday next for that purpose.

Princess Henry of Battenberg, Governor of the Isle of Wight, will lay the foundation-stone of the eleventh block of houses to be added to the Roya National Hospital for Consumption at Ventnor on Saturday, July 31st.

Birmingham is setting a good example in the facilities it affords its medical men. The Health Committee have arranged with the Mason University College to make bacterial examinations for medical men in cases of suspected diphtheria, free of charge, and to supply them, when necessary, with anti-toxin serum, also free of cost.

The dignity of a baronetcy has been conferred upon Sir James Reid, K.C.B., M.D., Physician in Ordinary to Her Majesty. Sir William MacGregor, K.C.M.G., Governor of British New Guinea, who is a doctor of medicine, has had the Companionship of the Bath conferred upon him.

Last Saturday, July 3rd, was observed as Hospital Saturday in the metropolis, and the usual collections were made in the streets. A scheme resembling that of the Prince of Wales's Hospital Stamp receipts was adopted in connection with it. Sixpenny pictorial receipts were issued in exchange for a donation of that amount.

The conversazione given by the Dean and Faculty of University College last week was a very brilliant entertainment. Many of the exhibits were most instructive and interesting, and the floral decorations of the Slade School were most beautiful. This gathering is one of the pleasantest of the kind held in the metropolis. The guests numbered some thousands.

We are always glad to note signs of activity and good organisation in connection with charitable institutions. The Honourable Sydney Holland, chairman of the London Hospital, has adopted an excellent method of bringing the governors of the institution into touch with proceedings in connection with it by the means of notice cards sent to all governors. Thus he invites attendance at the present time at the exhibit of the London Hospital at the Victorian Era Exhibition at Earl's Court. We feel sure that such a method of stimulating interest in the hospitals to which the public subscribe will prove very fruitful of good results.

Yellow fever seems to be showing some tendency to spread from Panama to the Atlantic and Pacific ports of the United States, According to the *Medical Record* of New York nearly every ship from Colon has had one or more deaths among the passengers. On June 13th the *City of Para* arrived in San Francisco with one case of yellow fever on board, two deaths having occurred during the voyage from the Isthmus. Panama, on the Pacific side, appears to be the centre of the epidemic. No cases have as yet appeared at Colon on the Atlantic side, the passengers who have been attacked having contracted the disease at Panama.

